# Behavioral Genetics of the Interactions between *Apis mellifera* and *Varroa destructor*

**DOI:** 10.3390/insects10090299

**Published:** 2019-09-16

**Authors:** Alexis Beaurepaire, Christina Sann, Daniela Arredondo, Fanny Mondet, Yves Le Conte

**Affiliations:** 1 Abeilles et Environnement, INRA Avignon, 84914 Avignon, France; 2 Institute of Bee Health, University of Bern, 3000 Bern, Switzerland; 3 Labogena, 78350 Jouy-en-Josas, France; 4 Instituto de Investigaciones Biológicas Clemente Estable, 11600 Montevideo, Uruguay

**Keywords:** coevolution, host-parasite interactions, behavioral genetics, molecular ecology, population genetics, reproduction biology, microsatellite markers, *Varroa*, *Apis*, honeybees

## Abstract

The western honeybee *Apis mellifera* exhibits a diverse set of adaptations in response to infestations by its most virulent disease-causing agent, the ectoparasitic mite *Varroa destructor*. In this study, we investigated the effect of honeybee pupae genotype on the expression of four host and parasite traits that are associated with the reproductive phase of the mite in the brood of its host. We first phenotyped cells containing bee pupae to assess their infestation status, their infestation level, the reproductive status of the mites, and the recapping of cells by adult workers. We then genotyped individual pupae with five microsatellites markers to compare these phenotypes across full sister groups. We found that the four phenotypes varied significantly in time but did not across the subfamilies within the colonies. These findings show that *V. destructor* mites do not differentially infest or reproduce on some particular honeybee patrilines, and that workers do not target preferentially specific pupae genotypes when performing recapping. These findings bring new insights that can help designing sustainable mite control strategies through breeding and provide new insights into the interactions between *A. mellifera* and *V. destructor*.

## 1. Introduction

Social insects are characterized by large numbers of individuals living in close proximity in a nest. Within this group, eusocial insects are defined by the presence of overlapping generations, cooperative brood care, and division of labor into reproductive and non-reproductive individuals. Despite their multiple advantages, these traits make eusocial insects particularly vulnerable to diseases [1]. In response to this increased susceptibility, a wide range of collective and individual defenses against parasites and pathogens have evolved in insect societies [2,3]. Within an eusocial insect colony, the diversity of resistance and tolerance traits can be very high, even more so if queens mate with several unrelated males [4,5,6].

Honeybees (genus *Apis*) stand amongst the most polyandrous eusocial insects [7]. In this taxon, diploid queens achieve extreme levels of multiple mating with males through highly diverse and dynamic lek-like mating systems called Drone Congregation Areas (DCAs) [8,9,10,11]. Colonies of honeybees are formed by a single polyandrous queen, which produces haploid males and diploid females that either turn into new queens or into workers. Within a nest, workers can be full-sisters if they share the same parents, or half-sisters if their fathers differ. In the Western honeybee, *A. mellifera*, about twelve half-sister groups (called “subfamilies” or “patrilines”) are found in average per colony [7]. In addition to these extreme levels of polyandry, very high rates of recombination have been documented across the genome of this species [12]. Altogether, this enhanced genetic diversity affects colonies development [13,14] and can increase chances of the colonies to survive diseases [15].

*A. mellifera* suffers from a great number of diseases, of which the varroosis is undoubtedly the most harmful at the global scale [16,17]. This syndrome is caused by the ectoparasitic mite *Varroa destructor* and generally leads to the rapid collapse of the western honeybee colonies if no treatments are applied. The native parasite of *Apis cerana* in Asia is a mite that has managed to spillover to *A. mellifera* after colonies of the western honeybee were introduced in its distribution range and subsequently spread across most regions of the globe [18]. Acting as a vector for highly virulent honeybee viruses [19,20,21], *V. destructor* particularly affects the brood of its host, where its reproduction takes place [22].

The life cycle of *V. destructor* is composed of two phases: reproduction within brood cells and dispersal on the adult workers and drones. Reproduction starts with the invasion of a 5th instar larvae brood cell by a mature female mite (the foundress) shortly before capping by the workers. Approximately three days after the cell is closed, the foundress will lay a first haploid egg, which will develop into a male. She will then lay several diploid female eggs in 30 h interval. Her offspring will take about six days to reach maturity. At this stage, reproduction will occur between the mature offspring in the cell, resulting in incestuous mating if the cell was infested by a single foundress, or in the possible admixture of mite lineages if several foundresses initially infested the same cell [23,24]. Mating will occur until the host is fully developed and emerges, after nine to twelve days post-capping. Once the fully developed bee exits the cell, the mated female mites will enter a dispersal phase. They will crawl on the combs, climb onto adult bees, and hide between the sclerites of their host until an opportunity to infest new brood cells emerges. *V. destructor* dispersal phase finishes with the detection and infestation of a new 5th instar larva cell, where a new reproductive cycle can start. A wide variety of factors are believed to trigger host finding, and chemical cues from the host seem to play a crucial role in this important step [25].

A single *V. destructor* foundress will typically perform several reproductive cycles during its life [26], leading to a rapid buildup of parasite populations within a honeybee colony [27,28]. However, the reproduction of *V. destructor* depends on the availability of bee brood, which fluctuates greatly during the season, across environments and among *A. mellifera* populations [29]. In addition, the type of brood (i.e., worker vs. drone) also affects mite population dynamics. In fact, the honeybee drone brood, which takes more time to develop and leads to the production of more offspring per foundress than the worker brood, is more attractive to *V. destructor* [30,31,32]. However, little knowledge exists on the links between the invasion behavior of the mite and the biology of the brood. More specifically, whether mites consider certain biological traits of the individual larva they infest is currently unknown.

The rapid growth of *V. destructor* populations in *A. mellifera* generally induces the collapse of colonies within a few years in the absence of beekeepers’ intervention. However, several resistance and tolerance traits against the parasite have arisen and some Western honeybee populations can now survive without treatments [33,34]. One of these adaptations, the Suppression of Mite Reproduction (SMR), is of particular interest for beekeepers, breeders and scientists [35,36]. This trait is highly heritable [37,38] and consists of the absence or the delay of mite foundresses’ egg laying in the host brood cells and results in strong diminution of the parasite population dynamics. However, the biological mechanisms behind SMR are currently not fully understood. More specifically, whether the reproduction failure of the parasite is solely due to the action of adult workers, of brood, or both simultaneously remains unclear. In fact, adult worker bees may interfere with the mite reproduction through a diverse range of mechanisms, including the detection, unsealing, and resealing of the infested cells (the “recapping behavior”), or the selective removal of the infested pupae (the “Varroa-Sensitive Hygiene”, or “VSH behavior”) [39,40,41,42]. Yet, the brood may also play a role by directly altering the reproduction of the mite with kairomones [43] or by signaling the workers that it is infested [44].

We herein used behavioral genetics to investigate the interactions between *A. mellifera* and *V. destructor*. This discipline aims at unraveling the links between behaviors and genotypes and has been used extensively to study *A. mellifera* [45]. In this study, we first investigated four traits of the mite or of its host that take place during the parasite reproductive cycle: the infestation of brood cells, the number of foundresses infesting brood cells, the reproduction of foundresses, and the recapping of cells by adult bee workers. We then compared these different phenotypes to the genotype of the brood on which they were observed, using sibship reconstruction analyses with microsatellite markers to reconstruct the subfamilies of the pupae. The comparison of the phenotypic traits across patrilines allowed us to investigate whether specific bee subfamilies (i) are more frequently targeted by varroa infestations, (ii) are able to block the reproduction of the mites and (iii) are more likely to be opened by workers performing the recapping behavior.

## 2. Materials and Methods

### 2.1. Phenotyping

Reproductive traits of *V. destructor* mites were analyzed in the worker brood of seven colonies of *A. mellifera* located in the apiary of the INRA institute of Avignon, France, from the end of August to the end of October 2018 (Table S1). These hives had not been treated against *V. destructor* for over a year before the start of the experiments. Brood cells that were at least seven days post-capping (i.e., purple eyes pupal stage) were carefully opened with insect tweezers and their content was examined with stereo microscopes. The examination of the brood cells consisted of: (i) observing whether the capping had been manipulated by the workers (“recapping behavior”), (ii) noting the age of the pupae (from day 7 to day 11 post-capping) following [31], (iii) counting the number of foundresses and (iv) carefully describing all other varroa stages found in the cells, according to the methods detailed in [46]. The recapping behavior was monitored by checking whether the silk that usually lines the underside of the cap was lacking (recapped cell) or not (untouched cell) following [39]. A successful reproduction was presumed if at least one offspring female had sufficient time to mate with her brother before their host’s emergence. In all other cases (e.g., no male or no offspring females in the cells, not enough time for reproduction before emergence, etc.), the reproduction was considered unsuccessful. Using these observations, the presence/absence of mites, the number of foundresses, the mite reproduction level and the recapping status of every cell were obtained. Colonies were screened every two weeks in order to assess the temporal variability of the traits. However, due to complications (e.g., requeening of colony A), each of the colonies could not be screened at all time points (Table S1). In all, 2627 cells were phenotyped in the seven colonies (Table S1).

### 2.2. Genotyping

After phenotyping the brood, the pupae were collected and a hind leg per individual was dissected and placed in a 96 PCR well plate containing 100 µL of 5% Chelex solution per well. The location of each pupa on the plate was recorded to be able to keep tracks of the phenotypes of the individual cells during the downstream analyses. Directly after sampling, 5 µL of proteinase K (10 mg/mL) was added to each well of the plates and DNA extraction protocols were run in a thermocycler according to [47]. The plates containing the DNA were then stored at −20 °C until further use.

To analyze whether the different phenotypes matched the workers’ genotypes, the individuals from three colonies (B, D and E, N = 556 pupae) were sent to Genoscreen (Lille, France) to be genotyped on a 3730 XL sequencer (Applied Biosystems^®^, Foster City, CA, USA) at five microsatellite markers [48,49] (Table S2) using a single marker per PCR reaction and following the standard conditions detailed in [50]. The three colonies used for genotyping were selected according to their level of variability of the phenotypes, the dates when they could be sampled, and the number of pupae collected. Once retrieved, the genotypic data were scored manually using Peak Scanner v. 1.0 (Applied Biosystems^®^). The genotyping process was repeated once per sample if the first PCRs did not work. After this, individuals with missing data were discarded, resulting in a final dataset including 486 individuals.

To test the independence of the microsatellite markers, linkage disequilibrium tests were run for each pair of loci on the overall dataset using the software FSTAT v. 2.9.3. [51]. The number of alleles and observed heterozygosity levels were then estimated for each locus in every colony and over the three colonies (Table S2) using the microsatellite toolkit Excel add-on [52]. To assess whether the markers used were variable enough to accurately discriminate distinct bee genotypes, the Non-Detection Error coefficient (*NDE*) was calculated according to [50]. This index is derived from the number and frequency of alleles at the markers used and represents a probability of non-discrimination between two different genotypes due to a lack of polymorphism in the marker set used.

### 2.3. Testing the Links between Phenotypes and Genotypes

To identify subfamilies in the colonies, the queen and drone alleles of the genotyped workers were identified based on the frequency and pattern of the alleles, following the guidelines from [50]. With this procedure, the patrilines of the workers in the three colonies were reconstructed. In addition, the Non-Sampling Error coefficient (*NSE*) was estimated for each colony in order to assess whether the sample size used in this study was large enough to accurately grasp the diversity of subfamilies in each colony. This second index takes into account the distribution of the individuals of each genotype and provides an estimate of the number of patrilines that have not been sampled [50].

After these controls were performed, statistical analyses were conducted. Four independent Generalized Linear Models (GLMs) were used to assess the effects of three factors on the four different phenotypes: the patrilines of the individual pupae on which the traits were recorded, the colony of origin, and the date of sampling. To do so, only patrilines with at least five individuals were kept in the dataset. This cutoff number was selected as it allows keeping a sufficient number of patrilines for the analyses while retaining enough of the samples to allow assessing the variance within subfamilies. Given that no patriline was found in two colonies simultaneously, we used a nested design to account for both the variability explained by the differences between colonies and by the patrilines within each of the colonies. For three traits (infestation status, reproduction and recapping) the family used was Binomial, and for the fourth (infestation level) a Quasipoisson family was used. These statistical analyses were performed in R v. 3.6.1. [53].

## 3. Results

### 3.1. Phenotyping

In all, 2627 cells containing at least seven days old worker pupae were analyzed in the seven colonies screened during the phenotyping step (Table S1). These phenotypic results were variable across colonies (Table 1). Notably, recapping was significantly correlated with the mite infestation level of the colonies (r^2^ = 0.396, *p* = 0.005). Yet, at the individual level, recapping was not systematically higher in infested cells (Table 1), indicating that they may not be preferentially targeted by workers performing this behavior.

### 3.2. Genotyping

Three colonies out of the seven phenotyped were genotyped. Highly significant genetic disequilibrium was found over all samples between each pair of markers, showing that the five markers were independent of one another. In addition, the Non-Detection Error of the microsatellite markers used was small (NDE = 0.25–1.70%), indicating a low probability of not being able to discriminate two individuals with different genotypes (Table 2). Overall, 57 patrilines were found in the 486 individuals genotyped. Moreover, the Non-Sampling Error was small in all three colonies (NSE = 1.10–2.44), indicating that the great majority of subfamilies were reconstructed. Interestingly, none of the patrilines was found in two colonies simultaneously. In addition, the subfamilies were found evenly across the different sampling dates (Table S3).

### 3.3. Association between Phenotypes and Genotypes

**Figure 1:**
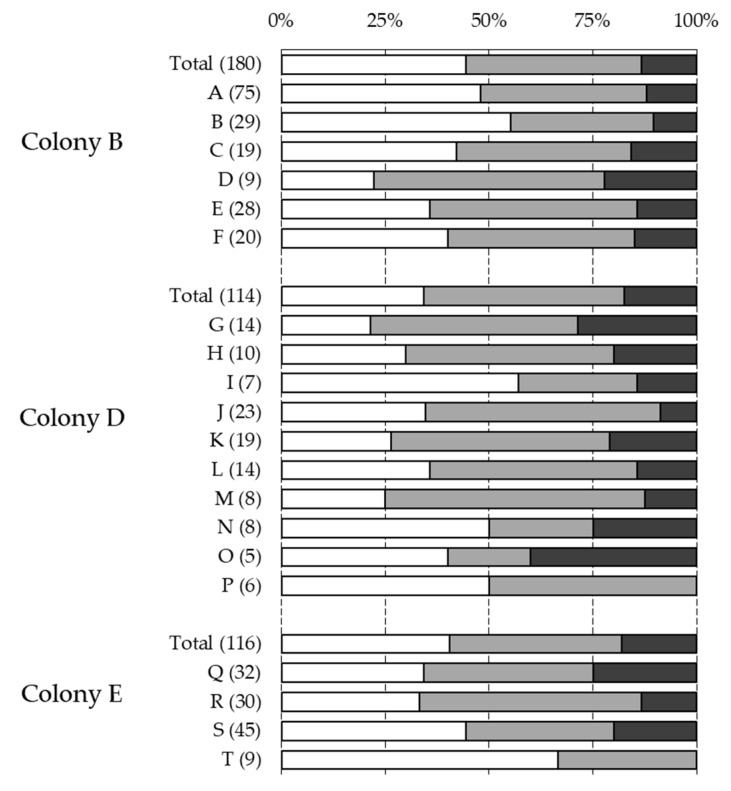
Association between Varroa infestation and pupae patrilines. Graph showing the proportion of brood cells that were non-infested (white), infested by one foundress (light grey), or by several foundresses (dark grey) associated with the different subfamilies of the three colonies genotyped. The total (Total) and patriline (letters) proportions are presented, with corresponding sample sizes indicated between parentheses. Only patrilines with five or more individuals are shown.

**Figure 2:**
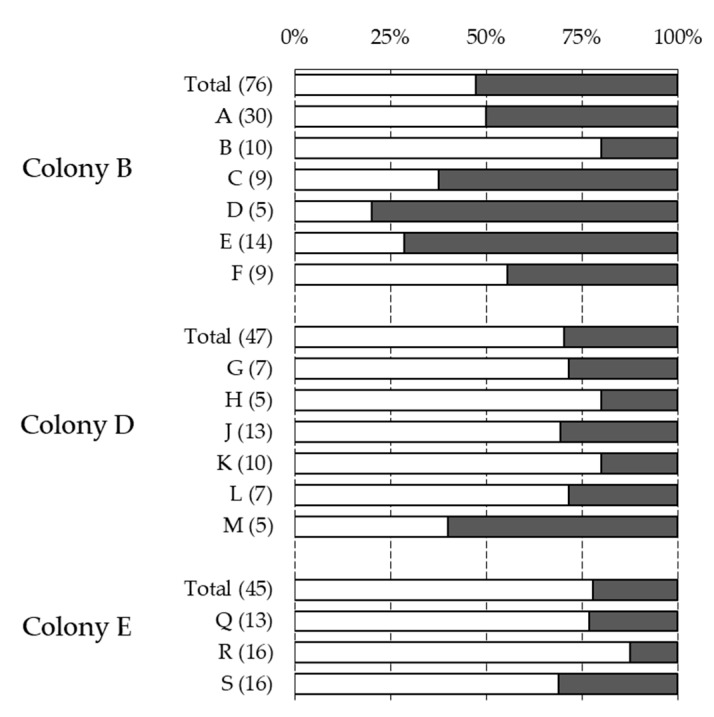
Association between Varroa reproduction and pupae patrilines. Graph showing the proportion of brood cells that contained a single non-reproducing foundress (white), or a single reproducing foundress (dark grey) associated with the different subfamilies of the three colonies genotyped. The total (Total) and patriline (letters) proportions are presented, with corresponding sample sizes indicated between parentheses. Only patrilines with five or more individuals are shown.

**Figure 3:**
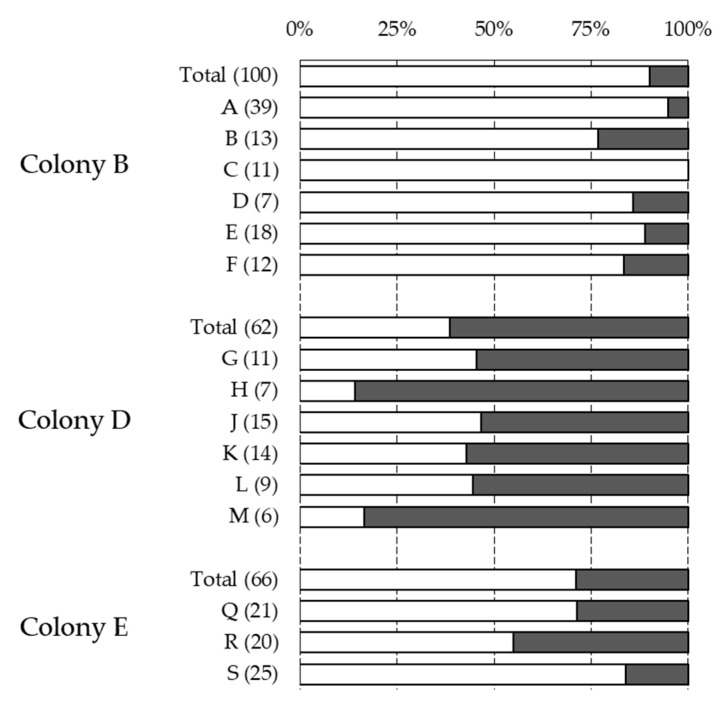
Association between cell recapping and pupae patrilines. Graph showing the proportion of non-recapped (white), or recapped (dark grey) brood cells associated with the different subfamilies of the three colonies genotyped. The total (Total) and patriline (letters) proportions are presented, with corresponding sample sizes indicated between parentheses. Only patrilines with five or more individuals are shown.

The statistical analysis using GLMs revealed that the honeybee subfamilies could not significantly explain the observed variability of the four phenotypes investigated (Table 3). Moreover, the expression of these traits across patrilines revealed no major deviation from the proportion of the phenotypes at the colony level (Figure 1, Figure 2 and Figure 3). Although the infestation status and infestation level did not vary significantly across the three colonies, mite reproduction and recapping did. Finally, all four traits were influenced significantly by the period of sampling (Table 3). In the three colonies sampled, the infestation status and level decreased slightly after the first sampling period (end of August) but increased notably towards the last period (end of October) (Table 1). A notable temporal decrease of mite reproduction was observed, whereas recapping showed an increased expression towards the end of the season (Table 1).

## 4. Discussion

In this study, we used behavioral genetics to investigate the interactions between *A. mellifera* and *V. destructor*, focusing on several crucial aspects of the reproduction of the mite. To do so, we compared the status and level of infestation of the mite, its fertility, and the recapping behavior of workers to the subfamilies of the pupae where these phenotypes were observed. Our results show that the traits varied in time, but revealed no significant associations between these phenotypic observations and the most prevalent patrilines found in the colonies we investigated, suggesting that these traits are not strictly determined by the genotype of the drones siring the honeybee worker brood.

Although not all colonies could be sampled at each time point, our data provide interesting insights into the temporal evolution of the expression of the traits we observed. In fact, the different phenotypes we investigated in this study varied significantly across the different sampling dates. First, the infestation level (number of infested cells and multiple infestations) of *V. destructor* over all colonies showed variation that reflect *A. mellifera* colony dynamics. In regions with a hot climate like Provence, where this study took place, *A. mellifera* queens typically stop producing brood during the hottest days of summer (mid-July to mid-August) and restart laying eggs again once the nectar flow restarts (end of August). In parallel, mite numbers generally increase in an exponential fashion throughout the brood season when no treatments are performed [28]. Our data reflect these population dynamics, since we found higher levels of infestation and multiple infestations at the end of August (i.e., moderate number of mites but few brood cells) and October (i.e., more mites and fewer brood cells) than in September (i.e., moderate number of mites but more brood). These findings are in line with previous studies documenting correlated population dynamics between brood and mites in *A. mellifera* colonies [24,27,28]. The next phenotype, the reproduction of *V. destructor*, varied greatly across the sampling dates and colonies and tended to decrease towards the end of the season. Temporal variation in *V. destructor* reproduction has been documented in the past [54,55], but these patterns may vary across years, and factors governing this variation remain currently unknown. Finally, the recapping phenotype varied highly across colonies, with a general temporal increase from the end of August to the end of October. In the recent past, this behavior has been proposed as a key mechanism explaining survival of mite infested colonies in the same honeybee population as we studied here [39]. However, although recapping significantly correlated with the level of infestation at the colony level, our data show that the expression of this trait on infested cells was not systematically greater than on non-infested cells. This result suggests that recapping does not take place in response to *V. destructor* infestation, and stress the need for more investigations on the mechanisms behind this behavioral trait. Overall, the important variations of the phenotypes we observed at the colony level may also be explained by the finite number of cells analyzed in this study. However, our aim here was not to provide precise colony-level parameters, but to look at individual bee phenotypes, an aim that was not perturbed by this phenotypic variation.

The genotyping revealed marked variation in genetic diversity across the three colonies. The number of markers and their polymorphism level, as well as the sample sizes used, permitted to study accurately the dominant subfamilies in the colonies, as reflected by the low *NDE* and *NSE* estimates. Notably, the distribution of the patrilines was homogenous across the collection dates, which is in accordance to former results on sperm admixture in the queen spermatheca [56]. Curiously, a consequent amount of rare subfamilies (<5 individuals per patriline) were sampled in the colonies. Unfortunately, these patrilines could not be included in our statistical analyses for methodological issues. Such rare patrilines have been documented in the past, and may have specific functions in the colony such as developing into emergency queens [57,58]. Although these subfamilies may possess increased resistance towards *V. destructor*, their low prevalence in the colony would not affect significantly the population dynamics of the mite, and the parasite populations could quickly build up on the brood of more frequent, sensitive patrilines. In contrast, some patrilines were very common in the colonies (e.g., 41.66% of individuals from the colony B belonged to a single patriline). Although this finding could be due to chance alone, it could also be caused by the fact that queens mated with several drones with identical genotypes (e.g., brothers from the same colony). Altogether, these observations stress the need for more studies on the colony-level behavioral genetics of *A. mellifera*, as little is currently known on the exact prevalence and specialization of the honeybee worker subfamilies.

These phenotypic and genotypic analyses allowed us to study the links between *A. mellifera* pupae subfamilies and several reproductive traits of *V. destructor*. First, the invasion behavior of *V. destructor* was not affected by its host subfamilies, since the presence/absence of mites and the number of foundresses did not vary significantly across bee patrilines. In the past, physical properties of the cells and the position of the larva have been shown to affect the mite invasion behavior [59]. In addition, mites use specific chemical cues of the larvae to infest cells [60] and mite infestation levels were shown to vary significantly between different bee brood race [59]. However, the distribution of mites in the brood cell of *A. mellifera* does not seem to reflect specific aggregation patterns [61]. Here, the absence of significant association between subfamilies and mite infestation bring new insights into the invasion behavior of *V. destructor* and adds to other recent findings showing that foundresses do not co-infest a cell based on genetic cues [23].

In parallel, the absence of significant association between the reproductive status of mites and the pupae subfamilies brings new knowledge on the expression mechanisms of SMR in diploid workers. The heritability of the main *V. destructor* resistance traits has been known for decades [37], and traits such as VSH have been used in selection programs with promising results. Notably, honeybee colonies selected for this trait also expressed lower mite reproduction levels [42]. SMR can be transmitted by queens to their progeny, and expressed in colonies even if the founding females are mated with unselected drones [62]. Interestingly, when performing crosses between colonies expressing high and low SMR levels, Locke [38] found that colonies formed with susceptible queen and resistant drones had low levels of mite reproduction, suggesting that SMR had a strong dominant genetic component that can be passed across generations by males. Our results do not match the predictions of that study. The variation of SMR was substantial in the colonies we genotyped (colony-level proportions of non-reproducing mites ranging from 0.46 to 0.20), but this trait did not vary significantly across subfamilies. These discrepancies may be due to the fact that SMR has a different genetic component in the colonies we studied compared to the Swedish resistant colonies analyzed by Locke [38]. Indeed, genomics studies performed in these two populations have found distinct genetic bases for this trait in Sweden [63] and in France [64]. However, the French *A. mellifera* population used in [64] is not the same as the population used here. Thus, conclusions from the latter study cannot be applied to our findings.

Finally, the recapping of cells by the adult workers was also not significantly affected by the subfamilies of the brood within the cells. Honeybee workers may be able to discriminate between brood genotypes, since behavior such as the rearing of emergency queens has been shown to be affected by the pupae’s subfamilies [57,58]. Our GLM also showed that recapping varied significantly in time and across colonies. While the reason for the temporal pattern we detected here remains unknown, high variability of this trait across colonies of Avignon and other populations was previously documented [39]. Here, irrespective of the fact that recapping has evolved in response to *V. destructor* in the population we studied (see above), our result indicate that the adult workers do not perform this behavior according to the brood subfamily found in the cell.

The level of *A. mellifera* genetic diversity has been linked to the level of resistance to *V. destructor* [65] and other pathogens [15,66] at the colony level. However, in this study we did not detect a significant link between the dominant honeybee worker subfamilies and the invasion behavior of the mite, or with the expression of two honeybee resistance traits (SMR and recapping). However, with our study design, we may have missed rare worker subfamilies that specialize in *V. destructor* resistance behavior. Although the expression of SMR in these rare patrilines would only poorly disturb the mite population dynamics at the colony level, workers from these patrilines could affect mite populations by specializing in recapping or other behavior such as Varroa-Sensitive Hygiene. The potential role of these rare subfamilies and the interactions between the different resistance traits at the colony level need to be further examined.

## 5. Conclusions

We have shown here that the dominant subfamilies of *A. mellifera* brood do not vary significantly in their attractiveness to *V. destructor*, do not distinctively impact mite reproduction, and are not differentially targeted by workers performing recapping behavior. While this work brings new insights into the co-evolution between the Western honeybee and its major parasite, our results also provide practical information for beekeeping. In fact, our findings suggest that two resistance traits believed to play a key role in the survival of *A. mellifera* colonies towards *V. destructor* infestation are recessive in the population we studied. Thus, breeding efforts relying on artificial insemination with sperm from resistant drones may only fail to produce colonies exhibiting the SMR and recapping traits. As this finding is in contradiction with others [38], further research should aim at comparing the genetic bases and inheritance mechanisms of these traits across *A. mellifera* populations to improve our current knowledge on this topic. This will surely help current breeding programs by allowing developing sustainable control strategies towards *V. destructor* to safeguard the Western honeybee’s valuable ecological and economical services across the globe.

## Figures and Tables

**Table 1:** Results of phenotyping. Details of the phenotyping of *A. mellifera* colonies showing the colony identification (Colony), the sampling period (Sampling; I: end of August, II: middle of September, III: beginning of October and IV: end of October), the number of infested cells (N_Inf_), the percentage of multiply-infested cells based on the total number of cells infested (% Multi), the proportion of mites reproducing (Reproduction), the percentage of cells recapped (% Recapped), and the percentage of infested cells that were recapped (% Inf Recapped).

Colony	Sampling	N_Inf_	% Inf	% Multi	Reproduction	% Recapped	% Inf Recapped
A	I	22	26.51%	9.09%	0.65	16.87%	22.73%
	I	25	28.74%	20.00%	0.85	34.48%	36.00%
	II	25	17.86%	24.00%	0.47	15.71%	36.00%
B *	I	24	29.63%	16.67%	0.60	2.47%	8.33%
	I	27	34.18%	25.93%	0.55	10.13%	18.52%
	II	28	18.92%	28.57%	0.65	6.76%	14.29%
	III	39	11.08%	20.51%	0.42	10.23%	2.56%
C	I	29	30.85%	31.03%	0.65	23.40%	17.24%
	II	27	38.03%	25.93%	0.40	42.25%	70.37%
	III	41	31.06%	29.27%	0.55	53.03%	36.59%
D *	I	28	21.71%	28.57%	0.85	6.98%	14.29%
	II	20	19.05%	0.00%	0.85	12.38%	0.00%
	IV	59	40.14%	32.20%	0.75	35.37%	59.32%
E *	II	22	23.16%	9.09%	0.90	13.68%	9.09%
	IV	33	54.10%	39.39%	0.65	90.16%	90.91%
	IV	58	38.41%	32.76%	0.69	82.78%	100.00%
F	III	45	13.80%	26.67%	0.42	28.53%	11.11%
	III	53	15.32%	20.75%	0.55	47.69%	50.94%
**Total**		**605**	**23.03%**	**25.12%**	**0.63**	**29.27%**	**38.84%**

* colonies selected for genotyping.

**Table 2:** Results of Genotyping. Details of the colonies genotyped. The name of the colony (Colony), the Non-Detection Error coefficient (NDE), and the total number of bees genotyped (N_Total_) are given, together with details on the patrilines (N_IND_: number of individual with data at all five markers, N: total number of patrilines, N > 5: number of patrilines with at least five workers, NSE: Non-Sampling Error coefficient).

Colony	NDE	N_Total_	Patrilines			
			*N_Ind_*	*N*	*N* > *5*	*NSE*
Colony B	0.71%	213	202	18	6	2.44
Colony D	0.25%	179	149	25	10	1.10
Colony E	1.70%	164	135	13	4	1.45
Overall	0.12%	556	486	57	20	5.52

**Table 3:** Results of the statistical analyses. Outputs of the four GLMs used to compare the effect of the colonies, patrilines and sampling date on the phenotypic traits studied (infestation status, infestation levels, reproduction and recapping). Significant *p*-values are indicated in italics and bold.

Model	Factors	d.f.	Deviance	Resid. Dev.	*p*-Values
Infestation Status	Colony	2	3.060	550.39	0.216
	Colony:Patriline	17	13.468	528.32	0.704
	Date	3	8.598	541.79	*0.035*
Infestation Level	Colony	2	4.2518	429.47	0.096
	Colony:Patriline	17	14.309	396.26	0.540
	Date	3	18.898	410.57	<*0.001*
Reproduction	Colony	2	10.086	211.53	*0.006*
	Colony:Patriline	13	14.310	187.90	0.352
	Date	3	9.315	202.21	*0.025*
Recapping	Colony	2	69.684	227.01	<*0.001*
	Colony:Patriline	13	14.507	133.94	0.269
	Date	3	78.564	148.45	<*0.001*

## Supplementary Materials

The following are available online at https://www.mdpi.com/2075-4450/10/9/299/s1: Table S1. Phenotyping details; Table S2. Information on the microsatellite markers used; Table S3. Prevalence of pupae patrilines across sampling dates.
