# Supplementary material for: Behavioral Genetics of the Interactions between Apis mellifera and Varroa destructor
Source: Insects. 2019 Sep 16;10(9):299. doi: 10.3390/insects10090299 (PMC6780334; doi:10.3390/insects10090299)
Supplement: Supplementary file 1 [file insects-10-00299-s001.zip › insects-573557-SI.pdf]

**Table S1.** Phenotyping details

Details of the sample size for the phenotyping conducted for this study. The colony name, date of samplings for each colony (Sampling; I: end of August, II: middle of September, III: beginning of October and IV: end of October), the number of cells analyzed (Ncells) and sampled (the number of non-infested cells N-I, cells with successful reproduction N-R, non-successful reproduction N-NR and multiple infestations N-MI) are given, as well as the sum of brood sampled per date and colonies. The stars next to the colony names indicate the colonies used for genotyping.

| Colony | Sampling | Ncells | N-I | N-R | N-NR | N-MI | Total sampled | |
|---|---|---|---|---|---|---|---|---|
| A | I | 83 | 24 | 19 | 1 | 1 | 45 | 143 |
| | I | 87 | 24 | 18 | 2 | 5 | 49 | |
| | II | 140 | 24 | 18 | 2 | 5 | 49 | |
| B* | I | 81 | 24 | 12 | 8 | 4 | 48 | 213 |
| | I | 79 | 24 | 11 | 9 | 7 | 51 | |
| | II | 148 | 24 | 13 | 7 | 7 | 51 | |
| | III | 352 | 24 | 13 | 18 | 8 | 63 | |
| C | I | 94 | 24 | 17 | 3 | 12 | 56 | 173 |
| | II | 71 | 24 | 20 | 0 | 8 | 52 | |
| | III | 132 | 24 | 15 | 14 | 12 | 65 | |
| D* | I | 129 | 24 | 17 | 3 | 8 | 52 | 179 |
| | II | 105 | 24 | 17 | 3 | 0 | 44 | |
| | IV | 147 | 24 | 24 | 16 | 19 | 83 | |
| E* | II | 95 | 24 | 18 | 2 | 2 | 46 | 164 |
| | IV | 61 | 24 | 13 | 7 | 13 | 57 | |
| | IV | 151 | 24 | 20 | 5 | 12 | 61 | |
| F | III | 326 | 48 | 12 | 19 | 13 | 92 | 167 |
| | III | 346 | 24 | 24 | 16 | 11 | 75 | |
| Total | | 2627 | 456 | 301 | 135 | 147 | 1039 | |

**Table S2.** Information on the microsatellite markers used

Details and references of the five microsatellite markers used in this study. The number of alleles (NA) and observed heterozygosity and their average value over all markers (+/- SD) are given for each colony and over all colonies (Total). The final sample sizes (i.e. after removing all individuals with missing information) for each colony and in total are given between brackets below the colony name.

| Name | Ref | NA | | | | HO | | | |
|---|---|---|---|---|---|---|---|---|---|
| | | *Col B (182)* | *Col D (130)* | *Col E (117)* | *Total (429)* | *Col B (182)* | *Col D (130)* | *Col E (117)* | *Total (429)* |
| A007 | [49] | 6 | 7 | 5 | 12 | 0.929 | 0.869 | 0.581 | 0.816 |
| Ap043 | [48] | 4 | 5 | 3 | 5 | 0.588 | 0.731 | 0.179 | 0.520 |
| Ap055 | [48] | 3 | 5 | 4 | 6 | 0.604 | 0.938 | 0.624 | 0.711 |
| A113 | [49] | 6 | 4 | 6 | 8 | 0.593 | 0.385 | 0.342 | 0.462 |
| B124 | [49] | 5 | 8 | 7 | 11 | 0.698 | 0.915 | 1.000 | 0.846 |
| AVG (SD) | | 4.80 (1.30) | 5.80 (1.64) | 5.00 (1.58) | 8.40 (3.05) | 0.682 (0.015) | 0.768 (0.017) | 0.545 (0.021) | 0.671 (0.010) |

**Table S3.** Prevalence of pupae patrilines across sampling dates

Details of the subfamilies sampled in this study, indicating the number of individuals per colony and patrilines across the sampling dates (I: end of August, II: middle of September, III: beginning of October and IV: end of October).

| Patriline | Colony | $N_{individual}$ | Date I | Date II | Date III | Date IV |
|---|---|---|---|---|---|---|
| A | B | 75 | 15 + 14 | 21 | 25 | - |
| B | B | 29 | 11 + 9 | 7 | 2 | - |
| C | B | 19 | 6 + 4 | 3 | 6 | - |
| D | B | 9 | 2 + 1 | 2 | 4 | - |
| E | B | 28 | 7 + 6 | 8 | 7 | - |
| F | B | 20 | 2 + 4 | 5 | 9 | - |
| G | D | 14 | 4 | 0 | - | 10 |
| H | D | 10 | 7 | 1 | - | 2 |
| I | D | 7 | 1 | 2 | - | 4 |
| J | D | 23 | 4 | 5 | - | 14 |
| K | D | 19 | 4 | 7 | - | 8 |
| L | D | 14 | 2 | 3 | - | 9 |
| M | D | 8 | 0 | 0 | - | 8 |
| N | D | 8 | 5 | 1 | - | 2 |
| O | D | 5 | 1 | 1 | - | 3 |
| P | D | 6 | 1 | 3 | - | 2 |
| Q | E | 32 | - | 14 | | 7 + 11 |
| R | E | 18 | - | 11 | | 0 + 7 |
| S | E | 31 | - | 8 | | 0 + 23 |
| T | E | 5 | - | 5 | | 0 + 0 |
